# Decomposing acute malnutrition by educational inequality of mother’s among under five children in Jammu and Kashmir

**DOI:** 10.1038/s41598-023-37587-y

**Published:** 2023-06-28

**Authors:** Insha Tariq, Javaid Iqbal Khan, Manzoor Ahmad Malik

**Affiliations:** 1 Department of Economics, University of Kashmir, Srinagar, Jammu and Kashmir 190006 India; 2 Department of Humanities and Social Sciences, Indian Institute of Technology (IIT), Roorkee, India

**Keywords:** Health care economics, Health policy, Paediatrics

## Abstract

Health outcomes in the state of Jammu and Kashmir have shown improvement in recent decades. However, nutritional achievements, particularly among children under the age of five, have not seen similar progress. Various factors influence the nutritional status of this age group, with the socio-cultural and biological attributes of mothers being considered significant determinants. While some studies have examined these attributes, there is a scarcity of research exploring the causal link between socio-culturally determined factors, such as maternal education, and child nutritional achievements, especially in Indian states located in North India. This paper aims to address this gap by analysing the prevalence of acute malnutrition (stunting) among children under five in Jammu and Kashmir in relation to educational inequality among mothers. The latest round of the National Family Health Survey (NFHS-5) is used to assess the levels of stunting (low height for age) among children, considering the literacy status of mothers and other control variables. Bivariate and multivariable methods are employed to study the association and identify risk factors. Additionally, the Oaxaca decomposition method is used to analyse the educational gap in factors associated with child stunting. The results indicate a higher prevalence of stunting among children of uneducated mothers (29%) compared to those of educated mothers (25%). The findings demonstrate a lower risk of stunting among children whose mothers are literate (OR 0.89). The Oaxaca decomposition analysis reveals a statistically significant difference in stunting between children based on their mother's education. These results highlight the wide disparities in acute malnutrition among children due to variations in maternal education. It is therefore crucial for policymakers to prioritize efforts to reduce educational disparities in order to alleviate the nutritional burden faced by children.

## Introduction

Education and nutrition are key components for overall wellbeing and contribute significantly to health and human capital^1^. Economic theories have established the role of better nutritional and educational outcomes in enhancing human capacities^2,3^. Therefore, these factors together are important for the overall growth of individuals. Inequalities in educational outcomes have a profound impact on health outcomes of populations at risk especially children^4^. Earlier studies have shown that children are at a greater risk of inhibited growth, multiple morbidities, and early death due to nutritional deficiencies^5–7^. Malnutrition retards physical and cognitive growth of a child and also impacts productivity in her later life^8^. However better nutrition can lower the risk of child morbidity and mortality^9^. Healthy children perform better on various wellbeing outcomes including educational attainment in later life^10^. Multiple individual and socio-demographic factors impact child malnutrition which include age of mother, birth order, gender of child, place of residence and wealth status^7,11,12^. Similarly, socio-geographical factors like caste, religion and region also account for a significant impact child malnutrition^13,14^. Various studies have identified these multiple factors determining acute malnutrition among the children. Individual and contextual factors like food insecurity, inadequate care, unhealthy environment, and mothers’ illiteracy result into acute malnutrition among the children^15–17^. These factors are pointedly related with child malnutrition but decomposition of these factors on key variables like education, particularly of the mother is scarce in emergent literature^4^. Although place of residence and wealth index has been studied to some detail, but very few studies have examined the nutritional deficiency of under five children across educational attainment of mothers^5,18^.

Malnutrition among under five children is an important social and public health concern in developing countries. Despite reduction in poverty levels and increasing economic growth millions of children under age five are suffering from nutritional deficiencies. Globally, malnutrition accounts for more than 30 percent deaths directly or indirectly among the under five children^19^. Around half of these deaths occur mainly due to acute malnutrition with children having low weight for height^20^. Therefore, children with stunting have a greater likelihood of death as compared to other nutritional deficiencies^21^. In India more than 30 percent of children suffer severe to acute malnutrition^22^. Although the prevalence has decreased over the decades but there is still a long way to go in terms of achieving the targets set by sustainable development goals.

Mothers’ education is attributed significantly to reduction of malnutrition among children^23^. Studies have shown that disparities in maternal education remain a key factor in determining stunting among the under five children^24,25^. However, intensity of the impact of mother’s educational attainment on child stunting in Indian settings has hardly been examined. Moreover, studies at regional level are extremely scarce despite the evidence that they do contribute significantly to child malnutrition in Indian^26^. Therefore, an informed understanding of the causal relationship between child stunting and differentials in mother’s educational attainment is inevitable for conversant policy intervention at the regional level. It is in this backdrop that the present paper studies determinants of child malnutrition by differences in educational attainment of mothers in Jammu and Kashmir. Estimates for contribution of various factors to stunting apart from mother’s educational attainment and differentials thereof are also provided.

## Data and methodology

### Methods

The study uses data from 5th round of nationally representative National Family Household Survey (NFHS-5) for the state of Jammu and Kashmir. Extracted data for 5731 under five children alongside their household attributes is used. NFHS employs a multi-scale stratified sampling design with households as the sampling unit. NFHS-5 sample is designed to provide estimates of all key indicators at the national and state levels, as well as estimates for most key indicators at the district level.

### Dependent variable

For estimation we first employed the Height for Age Z-score (HAZ) as a proxy for measuring stunting. HAZ below “minus two standard deviations” (− 2 SD) of the mean Child Growth Standards is categorized as stunted^27^.

### Exposure variable(s)

Mother’s education is an important determinant of child health. NFHS collects detailed information on mothers’ education. For the purpose of the present study mother’s education is divided into two categories: mothers with formal education (literate) and the illiterate. A set of individual characteristics such as age, gender, birth order and birth size are also used as control variables. Impact of socioeconomic factors like caste, religion, wealth, and region to on child stunting among under five children is also reported.

### Statistical analysis

Univariate and multivariate analysis was carried to study the results. Descriptive statistics are generated to present the distribution of respondents by educational differences of mothers. Binary logistic regressions are estimated to test for the association between the independent variables and the dependent variable. The logistic regression model estimates the probability of the dependent variable occurring as a function of the independent variables. It uses a logistic function, also known as the sigmoid function, to transform a linear combination of the independent variables into a probability value between 0 and 1. 0 represents children with no stunting whereas 1 represents children with any level of stunting in our analysis. The results were reported in odds ratio with along with confidence intervals. Odds ratio provides a measure of the change in the odds of the outcome for a one-unit change in the independent variable while holding other variables constant. Blinder-Oaxaca decomposition technique is used to decompose differences in stunting among under five children belonging to literate and illiterate separately: (a) to explain differences in the individual and socioeconomic characteristics of the children (distribution effect) and (b) to account for the differences in the effects of these characteristics (structure effect) on HAZ^28^. The Oaxaca decomposition analysis is a statistical method used to examine the contribution of different factors to observed group differences. It is typically used to understand the factors that contribute to disparities in outcomes between two groups.

## Results

**Figure 1:**
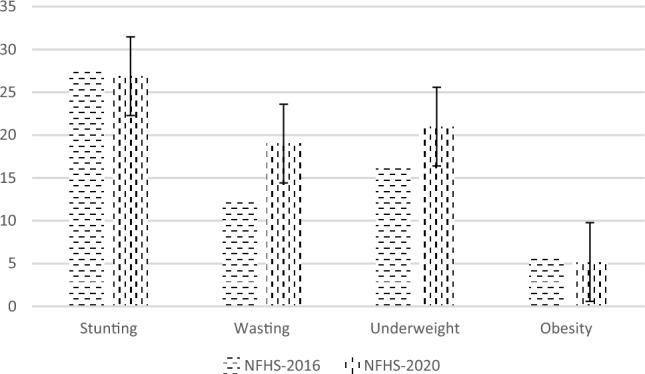
Trends in nutritional indicators among under-five children in Jammu and Kashmir.

Different indicators of malnutrition among under five children in Jammu and Kashmir are presented in Fig. 1. The stunting level in the state of Jammu and Kashmir has declined by less than 1 percent in the last five years. However, 27 percent of under-five children are still stunted. 19% of the children suffer wasting while as the proportion of underweight and obese are 21 and 5.2% respectively.

**Figure 2:**
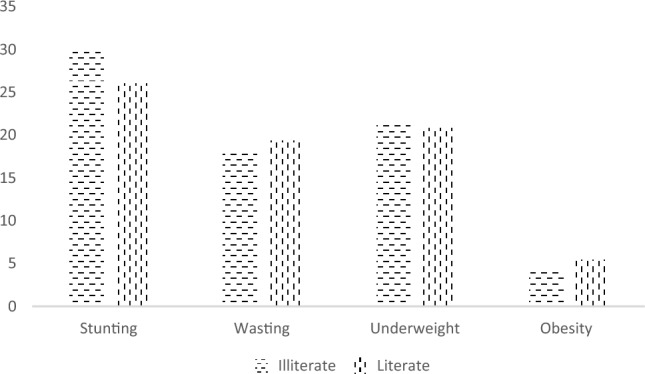
Nutritional status by educational outcome of mothers among under five children in Jammu and Kashmir- NFHS-5.

Figure 2 reports the nutritional gap by literacy among under five children. Nutritional gap for education is highest in stunting among under five children (at around 4%). 26 percent children whose mothers are literate are stunted, whereas this percentage stands at 30 percent for children whose mothers report no formal schooling.

Table 1 shows prevalence of stunting among children by educational difference of mothers along with other background characteristics. Stunting is highest among children of illiterate mothers across different background characteristics. 30.38 percent of all children aged 36–59 months are stunted; 37% male children belonging to illiterate mothers are stunted as compared to 28% children whose mothers possess any formal schooling. Significant differences are reported across individual characteristics like birth size and birth order. Overall prevalence of stunting is 31% in rural and 29% in urban children of illiterate mothers. Whereas 29% of the rural and 24% of the urban stunted children have literate mothers. Similarly, Variation in stunting among children by educational differences of mothers are observed across other background variables like social group, religion, and wealth index in Table 1 respectively.

**Table 1:** Prevalence of stunting by demographic and socio-economic characteristics with educational differences of mothers among under five children in Jammu and Kashmir NFHS-5.

Background characteristics	Stunting	Illiterate^a^	Literate^a^	Sample
Age of children				
0–11 months	28.67	29.10	28.56	1040
12–35 months	25.66	29.74	24.56	2208
36–59 months	27.28	30.38	26.31	2467
Sex of child				
Male	30.43	37.13	28.56	2903
Female	23.23	22.75	23.36	2828
Birth size				
Above average	23.28	21.4	23.74	662
Average	27.32	30.99	26.28	4600
Below average	26.31	25.98	26.41	361
Birth order				
Upto one	27.44	29.07	27.19	2397
2–3′	26.06	29.56	24.92	2952
4 and above	29.77	32.86	24.77	387
Residence				
Rural	30.12	31.00	29.98	4786
Urban	25.89	29.79	24.61	948
Social group				
Schedule caste	31.22	38.74	29.64	9.63
Schedule tribe	28.29	27.66	28.63	547
Other backward class	28.17	30.30	27.28	744
General	25.70	29.06	24.87	3888
Religion				
Hindu	27.01	36.30	26.02	1356
Muslim	26.33	28.63	25.47	4234
Other	42.20	67.78	39.38	132
Region				
Kashmir	26.25	27.92	25.62	1803
Jammu	27.48	33.24	26.32	2928
Wealth				
Poorest	31.31	33.01	29.48	866
Poorer	27.47	28.72	26.71	1167
Middle	26.44	32.48	24.62	1209
Richer	25.28	25.74	25.21	1401
Richest	26.07	14.06	26.39	1088
Total	26.89	29.96	25.99	5731

Authors own computation from NFHS-5.

^a^All values were significant at 5 percent level of significance using chi square test.

Table 2 provides the results for predictors of stunting among under five children in Jammu and Kashmir. The results exhibit that girl are at lower odds of being stunted as compared to boys in the study sample. Similarly, it is clear from the results that children below average birth size is at higher odds of being stunted as compared to normal children. at the regional level children from Jammu are at lower odds of being stunted as compared to Kashmir. From the table it can be seen that women children with literate mothers are at lower odds of being stunted compared to those who are having illiterate mothers.

**Table 2:** Predictors of stunting among under five children in Jammu and Kashmir NFHS-2019-20.

	Odds ratio	Confidence intervals (CI-95%)	
		UL	LL
Age of child			
0–11 months^®^	1		
12–35 months	0.07	0.67	0.96
36–59 months	0.87	0.73	1.04
Sex of child			
Male^®^	1		
Female	0.69	0.61	0.78
Birth size			
Above average^®^	1		
Average	1.10	0.90	1.35
Below average	1.12	0.83	1.53
Birth order			
Up to one^®^	1		
2–3	0.96	0.84	1.09
4 and above	0.97	0.74	1.27
Residence			
Rural^®^	1		
Urban	0.89	0.74	1.06
Social group			
SC^®^	1		
ST	0.87	0.65	1.17
OBC	0.95	0.73	1.25
General	0.80	0.64	0.99
Religion			
Hindu^®^	1		
Muslim	0.83	0.69	1.01
Other	1.52	1.02	2.25
Region			
Kashmir^®^	1		
Jammu	0.97	0.82	1.14
Wealth index			
Poorest^®^	1		
Poorer	0.92	0.75	1.14
Middle	0.85	0.68	1.06
Richer	0.82	0.65	1.02
Richest	0.76	0.59	0.98
Mothers education			
Illiterate^®^	1		
Literate	0.89	0.76	1.05

Authors own computation from NFHS-5.

Stunting was outcome variable (Reference = No stunting (0) and 1 = Stunting), ^®^ denotes for reference categories. UL and LL are upper and lower limits, CI-95%) shows precision of odds ratio at 95%.

Estimates of decomposition analysis are presented in Table 3. It can be observed that the mean value of stunting among children with illiterate mothers is 0.28. It is 0.25 among children with literate mothers. Thus, the difference in stunting among children due to mother’s education is approximately 0.028. The differences were further decomposed into explained and unexplained components. Explained component (E) measures the contribution of differences in the characteristics of the children and mothers to the differences in stunting between literate and illiterate mothers. The contribution of the explained component is 26.48%. This means that differences in the endowments of children explain 26.48% of variations of delayed growth for the children belonging to literate and illiterate mothers. Similarly, factors other than the endowment of children explain 73.25% of differences in child’s delayed growth with differences in educational outcome of mothers.

**Table 3:** Decomposition of stunting by educational differences of mothers in Jammu and Kashmir NFHS-5.

Stunting: Z-score weight for age lower than − 2SD	Coefficients	Confidence intervals	
		Lower limit	Upper Limit
Average difference in illiterate	0.280	0.2547	0.3046
Average difference in literate	0.252	0.2380	0.2650
Total difference	0.028	− 0.0002	0.0565
Decomposition difference			
Explained	0.007	− 0.006	0.021
Unexplained	0.021	− 0.010	0.052
%Explained (E/L-I)			26.48
%Unexplained (UE/L-I)			73.52

**Figure 3:**
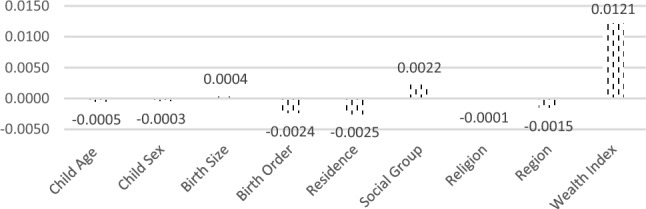
Contributions of differences in the determinants of stunting to the total gap between children belonging to illiterate and literate mothers in Jammu and Kashmir NFHS-5.

Figure 3 shows how differences in the distribution of each determinant contributed separately to explained difference. A negative contribution means that the determinant was narrowing the gap between children belonging to illiterate and literate mothers. Child age, gender, birth order, residence and region are reported to be most important. Contributions of the wealth index also is also reported to be significant at 5% level.

**Figure 4:**
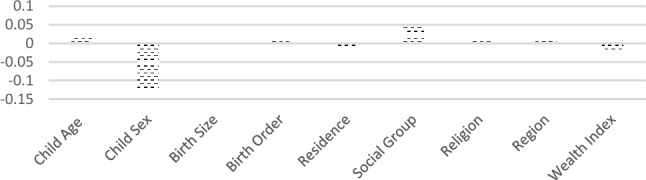
Contributions of differences in the effects of the determinants of stunting to the total gap between children belonging illiterate and literate mothers in Jammu and Kashmir NFHS-5.

Figure 4 exhibits detailed decomposition of the part of the gap that was caused by different effects of the determinants (factors other than the endowment of children). It shows the impact of factors like Child age, residence and wealth contributing significantly to the gap in stunting among under five children in Jammu and Kashmir.

## Discussion

This work tried to study the impact of mother’s educational differences on stunting among under five children in Jammu and Kashmir. Our results clearly demonstrate that children whose mothers have no formal schooling are at a greater risk of experiencing poor nutritional status. Using the Oaxaca technique, the study found that the difference in educational outcomes of mothers explains 26.48 percent of the variation in stunting among children under five.

Stunting is a major public health challenge affecting millions of children globally. Educational differences among mothers generate significant variations in and impact on child health outcomes^29^. Socioeconomic differences also account for significant changes in nutritional outcomes of the child^30^. This study provides empirical evidence of these differences, which are likely due to structural and compositional factors^4,31^.

The higher prevalence of stunting among children under five with illiterate mothers highlights the role of educational inequality in shaping stunting levels in Jammu and Kashmir. While this study is the first of its kind in the context of Jammu and Kashmir, studies across India have identified maternal education as a significant determinant of stunting, particularly when there are differences in income, social class, and work outcomes^18,26,28^.

Socioeconomic inequalities are evident at household level. Therefore, children belonging to poorer households, living in rural areas or other demographic characteristics of mothers and children result in lower intakes of nutrients, which therefore likely influences the stunting levels^32,33^. Thus, adjusting for these differences with educational outcome of mothers, the results show strong differences in stunting levels. Our results corroborate with earlier studies where mothers without having any formal schooling were associated with a higher stunting level among under five children^34^. Hence, greater educational attainment of mothers appears to have a mitigating impact on stunting levels among the under-five children. These differences in stunting are explained through a causal link between education of the mother and nutritional wellbeing of her child: higher levels of education can not only augment the ability of mothers to afford good nutrition for their children, but also enhances their awareness about the pros and cons of better nutrition^23^. Mother’s education also provides means to promote breastfeeding and equitable excess to nutritious foods which are critical in reducing the stunting levels as identified by earlier studies^35^.

Socio-economic and other factors are critical to increasing nutrition among the under five children across populations. While studying the role of these predictors separately, factors like child age, gender, birth order, religion and place of residence critically contribute to the nutritional deficiency among children. Our findings are therefore well in line with the earlier studies^36,37^.

Stunting is a major health challenge with significant impact on health and wellbeing of under five children. Educational inequalities have a strong likelihood of affecting the nutritional outcomes of children apart from other individual and contextual factors. The findings are consistent with the earlier literature where better socio-economic outcomes were associated with better nutritional outcomes^29,32^. It is therefore important that populations facing the stunting challenges with large gaps in mother’s education should focus on a new set of policy targets where not only the individual and socioeconomic characteristics of an individual are emphasized but also a strong focus is provided upon the compositional and structure effects that likely are key to nutritional variations across children.

## Conclusion

This paper examines the differences in educational outcome of mothers on stunting levels of their children in Jammu and Kashmir. The results reveal that most of the gap in stunting across different socio-economic and geographical factors are likely due to the unexplained contributions. However, distributional effect also implies a significant gap in stunting among the children belonging to mothers with educational inequalities^38^. The result clearly reflects upon the importance of mother’s education in nutritional deficiency and urges for strong policy intervention in this direction. To sum up this study decomposed the educational inequality in mothers to examine their impact on nutritional outcomes of children particularly stunting, the study clearly shows the immediate need for policy incentives to emphasis on the factors that can shape these determinants particularly with interventions focusing on the socio-economic characteristics of mothers.

## Data Availability

Dataset and codes used in this study can be accessed from corresponding author on request. The data is also available in public repository at www.dhsprogram.com.

## Abbreviations

NFHS: National family household survey
HAZ: Height for age Z score
SDG: Sustainable development goals
